# Evaluation of microstructural changes in spinal cord of patients with degenerative cervical myelopathy by diffusion kurtosis imaging and investigate the correlation with JOA score

**DOI:** 10.1186/s12883-020-01752-x

**Published:** 2020-05-13

**Authors:** Zhuohang Liu, Bingyang Bian, Gang Wang, Cheukying Tian, Zhenshan Lv, Zhiqing Shao, Dan Li

**Affiliations:** 1 Department of Radiology, The First Hospital of Jilin University, Changchun, Jilin 130021 People’s Republic of China; 2 Department of Orthopedics, The Third Hospital of Jilin University, Changchun, Jilin 130021 People’s Republic of China; 3 Icahn School of Medicine at Mount Sinai, New York, 10001 USA; 4 Department of Spine Surgery, The First Hospital of Jilin University, Changchun, Jilin 130021 People’s Republic of China

**Keywords:** Degenerative cervical myelopathy, Cervical spinal cord, Diffusion kurtosis imaging, Quantitative, Japan Orthopaedic association score

## Abstract

**Background:**

To explore the feasibility of the metrics of diffusion kurtosis imaging (DKI) for investigations of the microstructural changes of spinal cord injury in patients with degenerative cervical myelopathy (DCM) and the correlation between Japan Orthopaedic Association (JOA) scores and DKI metrics.

**Methods:**

Fifty-seven patients with DCM and 38 healthy volunteers underwent 3.0 T magnetic resonance (MR) imaging with routine MRI sequences and DKI from echo-planar imaging sequence. Based on the JOA score, DCM patients were divided into four subgroups. DKI metrics of the DCM group and control group were obtained and compared, separately for the white matter (WM) and the gray matter (GM).

**Results:**

The FA values in WM were significantly lower (*P* = 0.020) in the DCM group than in the control group. The MK values in GM were lower (*P* = 0.011) in the DCM group than in the control group. The MD values in WM were significantly higher (*P* = 0.010) in the DCM group than in the control group. In GM, the JOA score was positively correlated with the MK values (*r* = 0.768, *P* < 0.05). In the WM, the JOA score was positively correlated with the FA values (*r* = 0.612, *P* < 0.05).

**Conclusion:**

DKI provides quantitive evaluation to the characters of microstructure of the spinal cord damage in patients with DCM compared to conventional MR. MK values can reflect microstructural abnormalities of gray matter of the cervical spinal cord and provide more information beyond that obtained with routine diffusion metrics. In addition, MK values of GM and FA values of WM may as a be highly sensitive biomarker for the degree of cervical spinal cord damage.

## Background

Degenerative cervical myelopathy (DCM) is a common degenerative disease that causes cervical spinal cord (CSC) dysfunction. In this condition, osteophytes occurs during bone formation, disc degeneration and ligament hypertrophy compress or stimulate the CSC, which subsequently causes sensory and motor dysfunction or even paraplegia [1–3]. Conventional clinical magnetic resonance (MR) imaging often underestimates the degree of the damage of CSC lesions and is poorly associated with clinical severity. The absence of abnormal signal changes in the T2-weighted imaging during the early stage of the disease can lead and misdiagnosis or mistreatment [2]. Despite the clinical manifestations of the condition, no abnormal intensity is usually observed in the CSC in the conventional MR imaging during the early clinical stages. In fact, surgical indications for DCM patients remain to be established [4], but routine MR imaging such as T2-weighted imaging cannot find any abnormal signal intensity. Hence, a quantitative method for evaluating the microscopic damage of CSC in patients with DCM is highly required.

In addition to conventional MR imaging for observations of the morphological and signal intensity alterations of CSC, diffusion tensor imaging (DTI) has been recommended for assessing the microstructural changes in the CSC of DCM patients. DTI is based on the assumption that diffusing water molecules follows a Gaussian distribution, and DTI-derived quantitative parameters such as fractional anisotropy (FA) and mean diffusivity (MD). The FA values were most frequently used to measure diffusion anisotropy, and the MD values were considered to clearly reflect the molecular diffusivity under restricted motion. DTI was superior in observing the micropathology changes of neuro-degenerative disease over the routine MR imaging [5]. In patients with DCM, DTI metrics can be sued as an objective quantitative measurement methods to evaluating the microstructural pathology of the spinal cord [6, 7]. Increased MD and reduced FA values were previously observed at the compressed spinal cord regions, regardless of whether abnormal signal intensity in the spinal cord was detected or not on routine MR imaging [7]. However, in fact, the displacement of water molecule is restricted by barriers such as cell membranes and organecells in normal tissue. So, the assumption of Gaussian water diffusion may be inappropriate in biological structures. Moreover, DTI provides fewer valuable information in tissues such as gray matter (GM). This is because for the microstructure of GM is characterized as isotropic.

Diffusion kurtosis imaging (DKI) is a more advanced form of diffusion analysis, which does not require the assumption of a Gaussian shape and model of water molecules [8]. Therefore, it can more accurately reflect the micropathological abnormalities including injuries of neurons and nerve fibers and the therapeutic effects in neuronal diseases in vivo compared to DTI [9, 10], especially concerning the GM [11, 12]. DKI metrics provided a more comprehensive characterization and evaluation of the lesions and changes in the GM in the spinal cord of patients with multiple sclerosis [13]. Recent studies [14, 15] established that the mean kurtosis (MK) values provide additional information on the cervical spinal cord condition in patients with DCM.

The Japan Orthopaedic Association (JOA) score is a clinical index widely employed for evaluating the severity of cervical spinal cord injuries in patients with DCM. In the present study, our aims were the following: 1) to explore the feasibility of DKI metrics in evaluating the microstructural changes of CSC including the white matter (WM) and the GM, respectively, in DCM patients; 2) to assess the correlation between the JOA score and DKI metrics.

## Methods

### Subjects

Informed consent was obtained from all subjects included in the study, which was approved by our Institutional Review Board before study commencement.

From September 2017 to August 2018, 57 consecutive patients who had clinical signs and symptoms that indicated early-clinical-stage degenerative cervical myelopathy were enrolled in our study (DCM group). The DCM group consisted of 35 males and 22 females with a mean age of 48.21 ± 11.77 years old (age range 38–55 years). In addition, 38 healthy adults, including 22 males and 16 females, mean age of 44.11 ± 13.99 years old (age range 36–49 years), were included in the study (Control group). In the healthy subjects, cervical spinal cord MR imaging revealed no abnormal signals for diseases affecting the nervous system. The following exclusion criteria were applied: (a) lesion associated with other intraspinal diseases; (b) cervical and spinal trauma; (c) history of neck surgery; (d) unsatisfactory image quality for calculating diffusion metrics.

The degree of disability assessed by JOA score in patients with DCM. The lower the score, the more obvious the impairment is. In our study, all DCM patients were divided into four subgroups based on their JOA score: 13–16 points were considered as mild damage, 9–12 points as moderate damage, 5–8 points as severe damage, and 0–4 points as serious damage.

### Image acquisition

All images were acquired by a 3.0 T MR scanner (Philips Ingenia; Philips Medical Systems, Best, The Netherlands) using an 8-channel head and neck coil. The examiner took selected the advanced mode and fixed both the sides of the subject’s head with homemade rice bags. The examinee was the supine position and informed not to move the body as much as possible, to breathe calmly, and avoid swallowing movements and cough to reduce motion artifacts. The scanning sequences included routine MRI sequences (fast-spoiled gradient echo scout, T1-weighted images (T1WI), T2-weighted images (T2WI), and T2 fast-recovery fast-spin echo) and DKI sequences. Spin echo sequences and fast spin-echo sequences were used to obtain sagittal and axial T1-and T2-weighted images. The imaging parameters of the sagittal images were as follows: repetition time (TR)/echo time (TE), **550**/6.4 ms for T1WI as well as 3263/95 ms for T2WI; echo-train length of 4 for T1WI and 36 for T2WI; section thickness/gap 3/0 mm; number of slices, 12 sections; field of view (FOV), 250 mm; and matrix 256 × 256. The following imaging parameters were adopted for the axial images: TR/TE, **400/**11 ms for T1WI as well as 3861/127 ms for T2WI; echo-train length of 5 for T1WI and 36 for T2WI; section thickness/gap of 3/0 mm; number of slices, 15 sections; FOV, 200 mm; and matrix, 512 × 512. The imaging parameters for DKI were as follows: TR/TE, 1100/101 ms; section thickness/gap 3/0 mm; number of slices, 18 sections; FOV, 80 × 80 mm; matrix, 512 × 512; Four *b* values including 0, 700, 1400, and 2100 s/mm^2^ with diffusion encoding in eight directions for every *b* value. The gradient length (δ) and time between the two leading edges of the diffusion gradients (Δ) were 9.8 and 44.1 ms, respectively.

### Image analysis

All DKI data were imported into the EWS workstation (Philips Medical Systems, Best, the Netherlands). DKI metrics included the FA, MD, and MK values. First, the FA and MD maps were calculated in the routine mono-exponential model. Second, the diffusion kurtosis was calculated on a voxel-by-voxel basis as described in previous papers [16]. Four *b* values (0, 700, 1400, and 2100 s/mm^2^) were used in the following equation:

$$ \ln\ \left[\mathrm{S}\left(\mathrm{b}\right)\right]=\ln\ \left[\mathrm{S}\ (0)\right]-b\ast {\mathrm{D}}_{\mathrm{app}}+1/6\ast {\mathrm{b}}^2\ast {{\mathrm{D}}^2}_{\mathrm{app}}\ast {\mathrm{K}}_{\mathrm{app}} $$

where D_app_ is the apparent diffusion coefficient for the given direction, and K_app_ is the apparent kurtosis coefficient that is dimensionless.

**Fig. 1:**
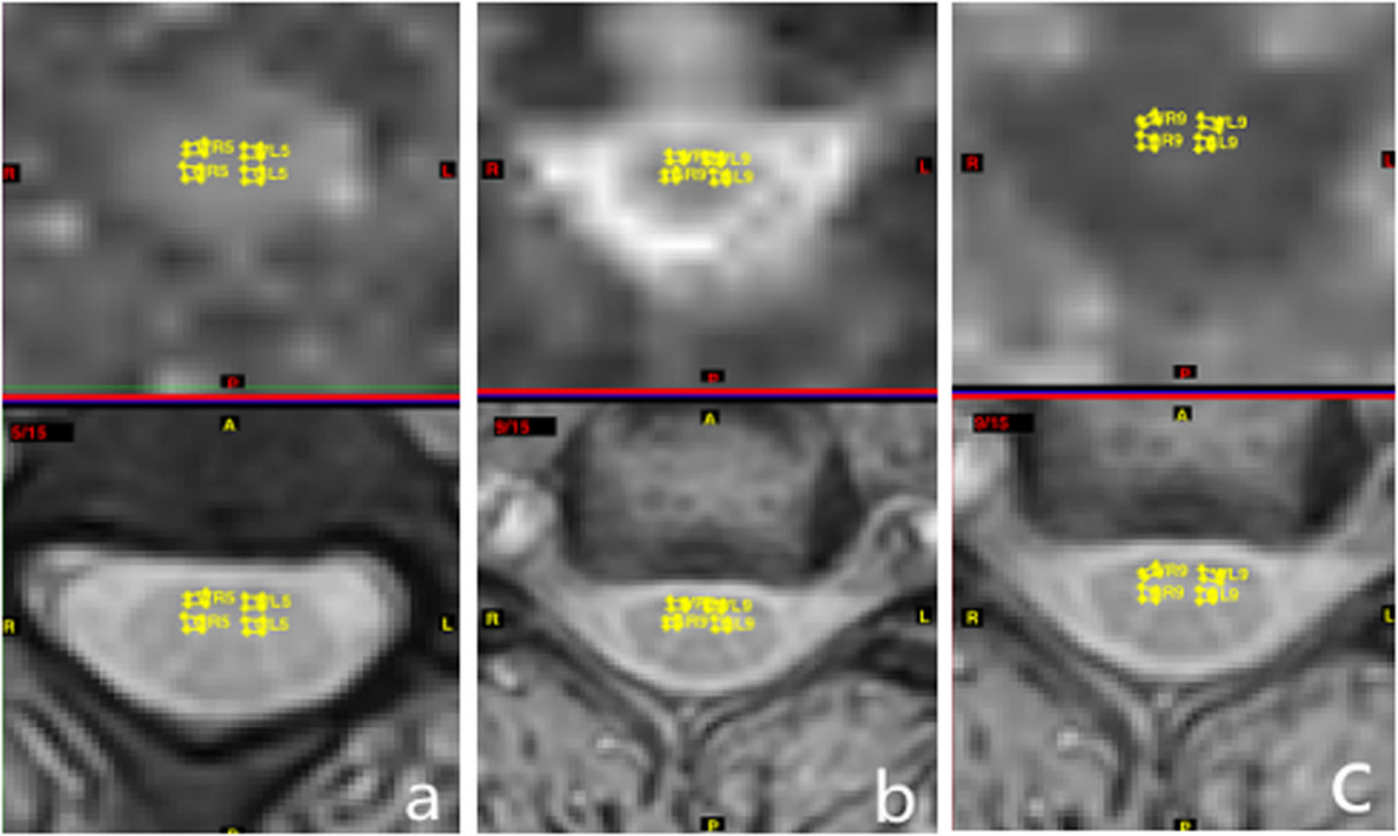
A healthy subject with T2WI image and corresponding metric maps of cervical spinal cord in the axial plane. Placement of ROIs in bilateral anterior horn of GM and anterior funiculus of WM and FA map (**a**), MD map (**b**), and MK map (**c**) at the C3–C4 vertebral level

**Fig. 2:**
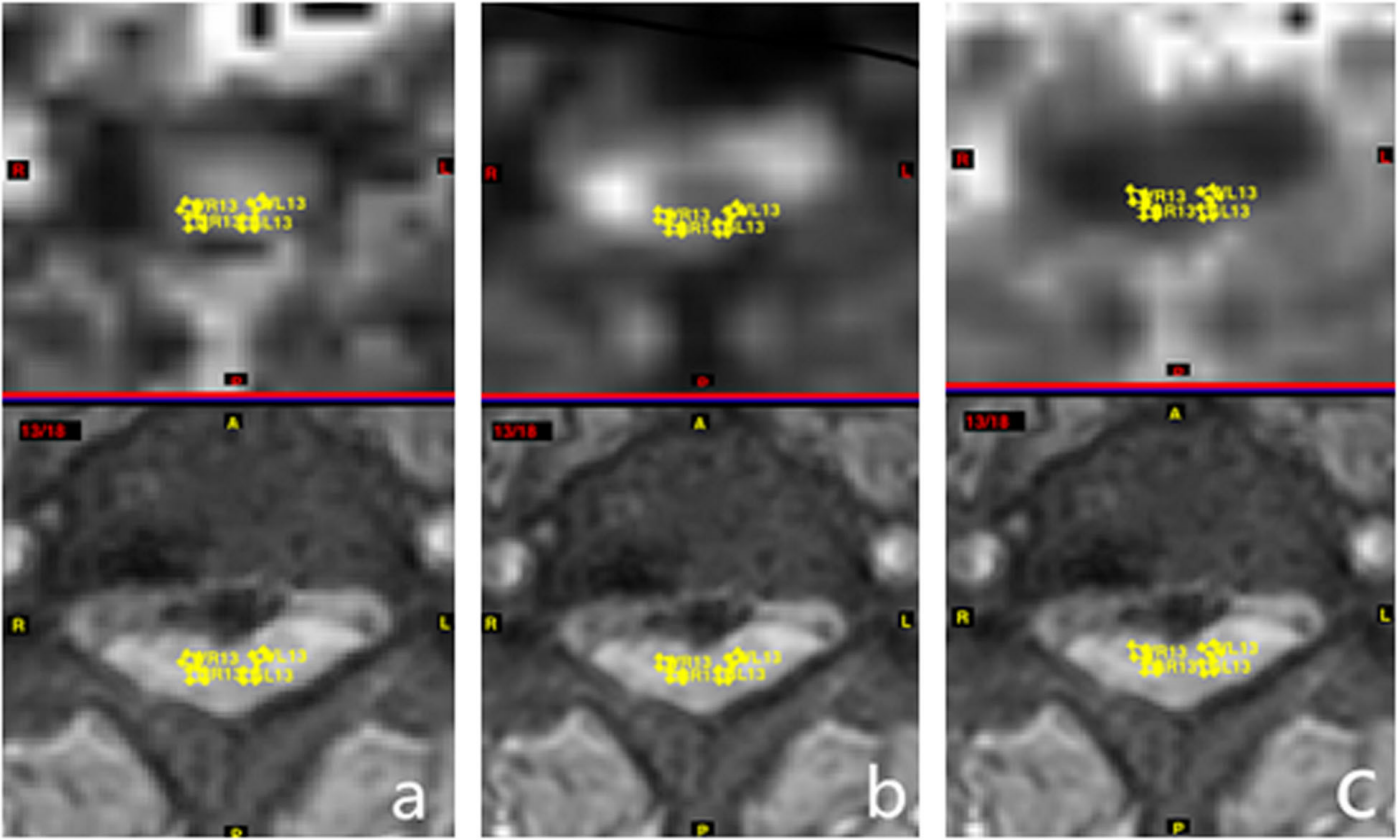
A patient with DCM with T2WI image and corresponding metric maps of cervical spinal cord in the axial plane. Placement of ROIs in bilateral anterior horn of GM and anterior funiculus of WM and FA map (**a**), MD map (**b**), and MK map (**c**) at the C3–C4 vertebral level

Based on the sagittal T2-weighted imaging findings, appropriate axial T2-weighted imaging was selected and utilized as an anatomic reference; an identical size (2 mm^2^) of a region of interest (ROI) was drawn manually on the spinal cord. The ROIs included both the bilateral anterior horn of GM and the anterior funiculus of WM. To reduce the partial volume effects and artifacts caused by the flow of the cerebrospinal fluid, the data within the WM had to be measured as close to the inner side of the spinal cord as possible and had to be distinguished from the GM. To ensure data consistency, the size and pixels of each ROI were not changed. These measurements were performed at five disc levels (from C2/3 to C6/7). The TEMINAL software allowed for copying of the ROIs and guaranteed the evaluation of the same region with diffusion metric maps in the control and the DCM group (Figs. 1 and 2). FA, MD, and MK values were measured in each area. Each measurement of ROI was repeated three times and completed by two neuroradiologists who were blinded to the patients’ individual information.

### Statistical analysis

SPSS-22.0 software ((SPSS Inc. Chicago, IL, USA) was employed for statistical analysis. An initial analysis was performed using the Anderson–Darling test to evaluate whether the data were normally distributed. Numerical data were expressed as means ± standard deviation (SD) and compared by Mann–Whitney U test or ANOVA, as appropriate. The correlation between JOA score and DKI parameters was calculated by the Spearman’s or Pearson correlation method. The consistency of the analysis results of two radiologists was checked by Kappa-test. The *K-*value > 0.6 was good, 0.4 ≤ *K-*value ≤0.6 was moderate, and the *K-*value < 0.4 was poor. *P* < 0.05 was considered to indicate statistically significant difference.

## Results

Table 1 shows demographics and clinical information. JOA scores were statistically different among the four subgroups. Three patients were excluded because of spinal canal meningioma (two patient) and syringohydromyelia (one patient).

**Table 1:** Main characteristics of 4 subgroups in DCM patients

Parameter	Mild	Moderate	Severe	Serious	*P-*values
Sex					0.805
Men	5	9	13	8	
Female	4	5	6	7	
Mean age(y)	44.67±13.32	47.43±13.01	50.05±10.64	48.73±11.66	0.722
Median JOA score (range)	15 (13–15)	10.5 (9–11)	6 (5–8)	3 (1–4)	<.001
Mean no. Of cervical spinal cord	0	0	0	0	**___**

DKI images were successfully obtained in all the patients. The Kappa value of the analysis results of the two radiologists was *K* = 0.741. The mean MK and FA values (both unitless) and MD values (× 10^^− 3^ mm^2^/s) of GM and WM ROIs compared at the C2–3 to C6–7 level in healthy subjects and patients with DCM without visible lesions are reported in Table 2. In patients, normal-appearing WM FA and normal-appearing GM MK values were significantly decreased (respectively, *P* = 0.002 and *P* = 0.011), and WM MD values were increased (*P* =0.010) compared with healthy controls while WM MK, GM FA and GM MD values were not significantly different (respectively, *P* = 0.078, *P* = 0.061 and *P* = 0.096) across the 2 groups of subjects.

**Table 2:** The mean of DKI metrics in the white matter and gray matter in DCM group and control group

Metrics	WM		*P-*values	GM		*P-*values
	CSM group	Control group		CSM group	Control group	
FA	0.710±0.087	0.769±0.088	0.002	0.555±0.091	0.616±0.074	0.061
MD	1.196±0.074	1.162±0.082	0.010	1.145±0.085	1.110±0.071	0.096
MK	1.105±0.102	1.159±0.111	0.078	1.098±0.087	1.150±0.105	0.011

*DCM* cervical spondylotic myelopathy, *GM* gray matter, *WM* white matter, *FA* fractional anisotropy, *MD* mean diffusivity, *MK* mean kurtosis. Units for FA and MK are dimensionless. MD values are given in 10^−3^ mm^2^/s

**Fig. 3:**
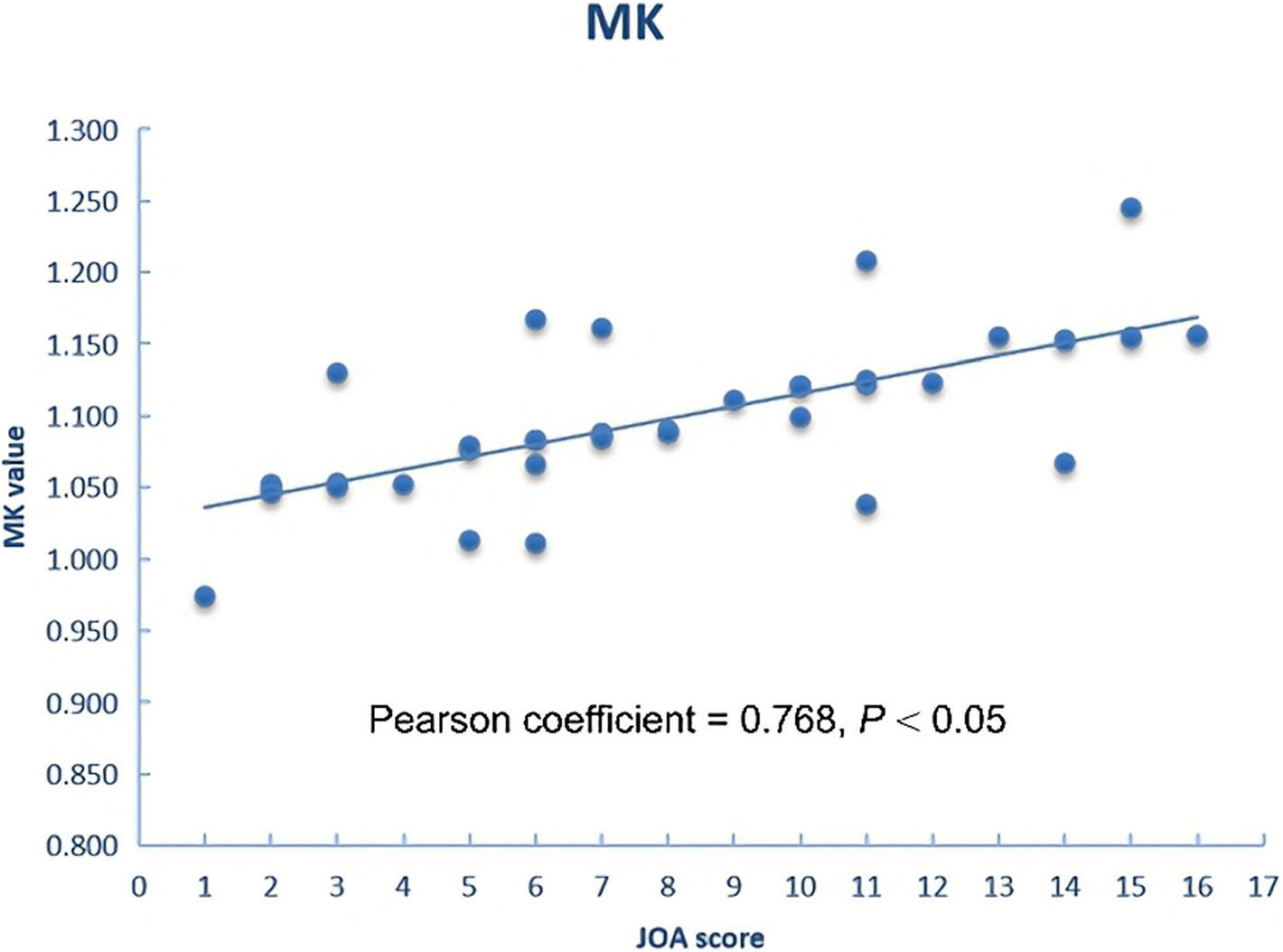
In GM, MK value is positively correlated with the JOA score in DCM patients

**Fig. 4:**
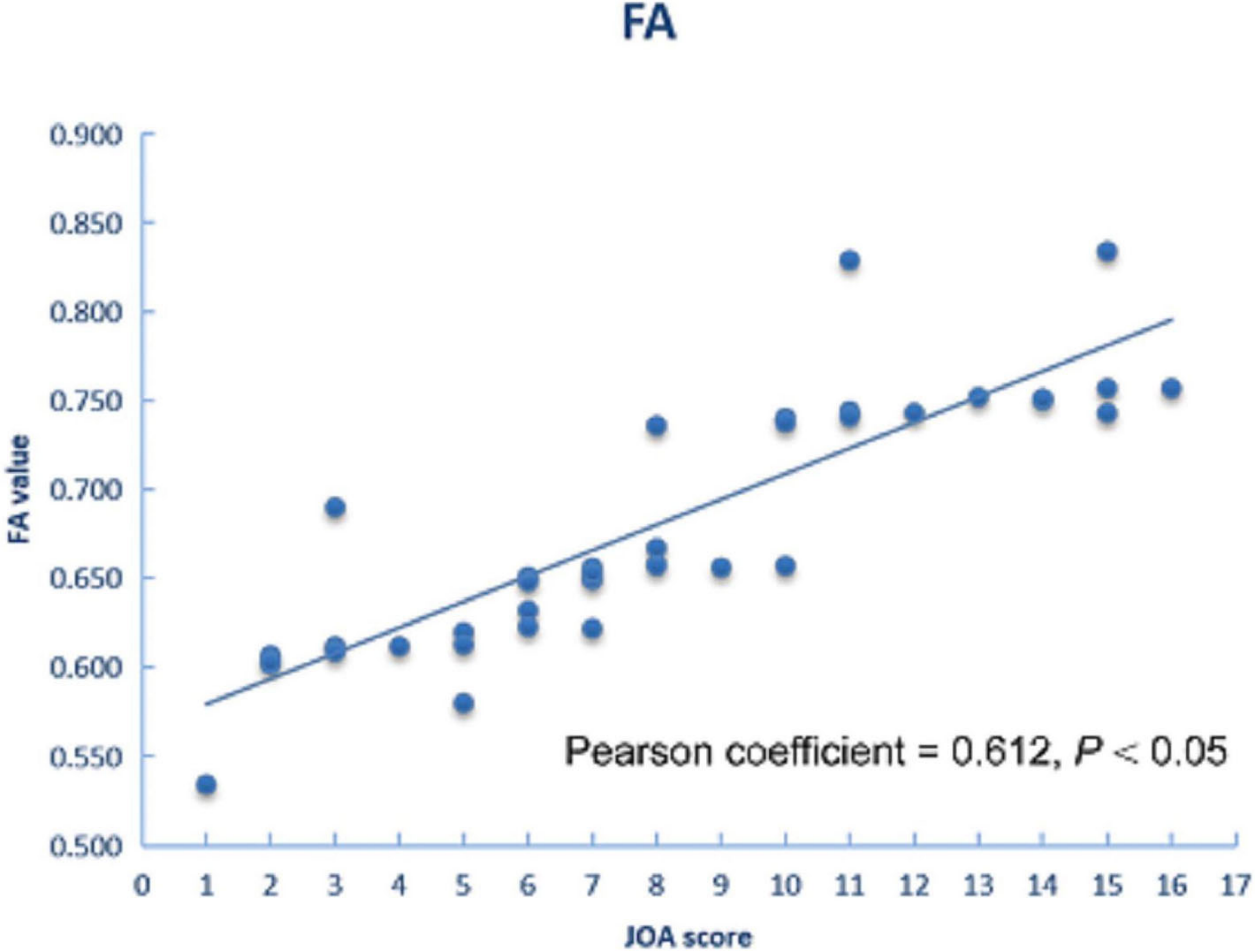
In WM, FA value is positively correlated with the JOA score in DCM patients

The JOA score and the DKI metrics of WM and GM in the DCM patients in the four subgroups were presented in Table 3. The relationship between the JOA score and DKI metrics is illustrated in Figs. 3 and 4. In GM, the JOA score was positively correlated with the MK values(*r* = 0.768, *P =* 0.018). In WM, the JOA score were positively correlated with the FA values(*r* = 0.612, *P* = 0.003).

**Table 3:** The mean of the DKI metrics in the area of white matter and gray matter in DCM patients with a different JOA score

Group	JOA score	Number	WM			GM		
			FA	MD	MK	FA	MD	MK
Mild	14.5±1.74	**9**	0.757±0.077	1.132±0.049	1.168±0.106	0.603±0.075	1.039±0.062	1.156±0.089
Moderate	10.5±1.98	**14**	0.743±0.086	1.190±0.046	1.163±0.104	0.607±0.092	1.106±0.067	1.123±0.085
Severe	6.5±1.54	**19**	0.703±0.082	1.167±0.074	1.060±0.084	0.509±0.080	1.166±0.059	1.090±0.077
Serious	2.5±1.56	**15**	0.658±0.078	1.253±0.085	1.072±0.082	0.537±0.081	1.219±0.053	1.052±0.078

*DCM* cervical spondylotic myelopathy, *GM* gray matter, *WM* white matter, *FA* fractional anisotropy, *MD* mean diffusivity, *MK* mean kurtosis. The units for FA and MK are dimensionless. MD values are given in 10^−3^ mm^2^/s

## Discussion

Conventional MR imaging methods such as T2-weighted imaging are widely used to assess the morphological changes in the cervical spinal cord in patients with DCM. However, there is a poor correlation between the MR findings and clinical symptoms, such as irreversible physical disability, likely due to the presence of microscopic injuries including demyelination, inflammation and axonal loss in the so-called normal-appearing GM and WM, not detectable by routine MR imaging [2]. In our study, T2-weighted imaging showed no abnormal signal intensity in the CSC of DCM patients, but the patient had clinical signs and symptoms that indicated degenerative cervical myelopathy. Therefore, observations of the micro-pathological changes in the CSC, such as axon loss and demyelination, are of great value in determining the treatment plans and evaluating prognosis, especially difficult cases. DKI parameters (FA, MD and MK values) accurately reflect the real situation of water molecule diffusion and provide additional and complementary information on the normal and pathological structural changes compared to routine MR in DCM patients [13, 17, 18]. Several reports have shown that reduced FA values and increased MD values in the CSC of patients with DCM in comparison with healthy controls [6, 7, 19]. Hori et al. [15] found FA values were significantly lower in compressed spinal cords than in uncompressed cords and MD values were not statistically significant. However, GM and WM were treated as a single unit when setting regions of interest (ROIs) and were not measured respectively in the above mentioned reports [6, 7, 15, 19]. So, this is likely to cause inaccuracy of DKI metrics. Our DKI metrics acquired GM and WM, respectively, showed extensive cervical spinal cord injuries compared to healthy subjects. In our research, we found that FA values of normal-appearing WM were significantly lower (*P* = 0.002) in DCM patients than in healthy controls, which is consistent with Li et al. [20]. Moreover, Li et al. [20] found no significant differences regarding MD values of WM between DCM group and control group. However, we research showed a significant increase of MD values in normal-appearing WM of DCM patients compared with controls (*P* = 0.010). The reduced FA values and increased MD values could be partly explained by the increased permeability of the membranes in the chronic hypoperfusion and disturbance of the arrangement of axons in the spinal cord [21].

DCM not only affects the WM, but also GM. DTI-derived metrics including FA and MD values have been shown to be sensitive to microstructural abnormalities of WM, but it is difficult to evaluate GM [20]. Some researchers [14, 15, 20] found that only the MK values of GM were statistical significant lower in the DCM patients than in healthy subjects. Their findings support the notion that the MK values are more advantageous over FA or MD values in evaluating GM injuries. In line with these findings, we found that only MK values of normal-appearing GM were obviously lower (*P* = 0.011) and there was no statistical difference in MD and FA values of GM between DCM group and control group, which further supports the notion that MK values of GM can provide information about the underlying tissue microstructure that is different and complementary to that obtained with DTI. A microcirculatory disturbance in the spinal cord may lead to lower MK values in GM [12, 16, 22, 23]. In addition, changes in the directions and components of the fiber tracts could have led to a decrease in the MK values [24, 25].

It is noteworthy that our study was the first to explore the correlation between the DKI metrics and the JOA score. In WM, FA values were sensitive to disease-related tissue injuries and was positively correlated with the JOA score (*r* = 0.612, *P* < 0.05). In GM, we found a significant correlation between the MK values of GM and JOA score (*r* = 0.768, *P* < 0.05), which suggests that the severity of CSC GM damage might have a role in the development of irreversible disability. The association between DKI metrics of CSC and JOA score indicated that WM-FA and GM-MK values could be used as an effective clinical aid to evaluate the physical function of patients with DCM. In clinical, routine MR imaging findings are not sufficient to warrant surgery or invasive therapy, but the relationship between DKI metrics and the JOA score can be a sufficiently precise and reliable approach for the assessment of DCM progression. Ideally, quantitative diffusion kurtosis image of CSC would be conventionally performed in clinical to monitor disease progression in DCM patients, but a lot of challenges must be overcome before translation from a study setting becomes feasible. At present, although there are relatively few researches of DKI applied to CSC in DCM patients, an appealing future direction for research efforts is brewing.

Overall, DKI may prove to be useful in the evaluation of the severity of CSC abnormality in DCM patients who demonstrate cervical spinal cord narrowing and symptoms without signal intensity change on routine MR imaging. In addition, this technique may be helpful in demonstrating early CSC damage in DCM patients, which may be related to transverse or longterm change. In clinical situations, one possible role for this type of imaging assessment would be to evaluate changes or progression as part of sequential follow-up of patients where the signs and conventional imaging findings are not sufficient to warrant surgery or invasive therapy but are sufficient to warrant follow-up.

## Conclusion

DKI of the cervical spinal cord can provide a quantitative way to assess microstructural characterization of normal-appearing WM and GM damage in DCM patients. Moreover, MK values can reflect microstructural change of gray matter of CSC and has the potential to provide new information prior to MD and FA values. MK values of GM and FA values of WM in the cervical spinal cord may as a be highly sensitive biomarker for the degree of CSC damage assessed by JOA score. Therefore, DKI should be considered as a reference for clinical therapeutic schedule and prognosis in DCM patients.

## Data Availability

The datasets supporting the conclusions of this article are included within the article. The raw data are not publicly available due it involves the privacy of patients but are available from the corresponding author on reasonable request.

## Abbreviations

DKI: Diffusion kurtosis imaging
DCM: Degenerative cervical myelopathy
CSC: Cervical spinal cord
WM: White matter
GM: Gray matter
MR: Magnetic resonance
JOA: Japan Orthopaedic Association
FA: Fractional anisotropy
MD: Mean diffusivity
MK: Mean kurtosis
